# Evaluation of Antiulcer Activity of 80% Methanol Extract and Solvent Fractions of the Root of *Croton macrostachyus* Hocsht: Ex Del. (Euphorbiaceae) in Rodents

**DOI:** 10.1155/2020/2809270

**Published:** 2020-04-09

**Authors:** Alefe Norahun Mekonnen, Seyfe Asrade Atnafie, Mohammedbirhan A. Wahab Atta

**Affiliations:** ^1^Department of Pharmacy, Teda Health Science College, Gondar, Ethiopia; ^2^Department of Pharmacology, School of Pharmacy, College of Medicine and Health Sciences, University of Gondar, Gondar, Ethiopia

## Abstract

**Background:**

Peptic ulcer disease causes significant mortality and morbidity. Plant kingdom provides a useful source for the development of new antiulcer agents. *Croton macrostachyus* is traditionally used to treat peptic ulcer in Ethiopia. This study aimed to evaluate the antiulcer activity of *C. macrostachyus* root extracts in rodents using different models.

**Methods:**

The crude extract was obtained by cold maceration in 80% methanol and fractionated with chloroform, ethyl acetate, and distilled water. The antiulcer activity was evaluated using pylorus ligation-induced ulcer model in Sprague Dawley rats and acidified ethanol-induced ulcer model in Swiss albino mice. The test groups received three doses (100, 200, and 400 mg/kg) of the crude extract and fractions for 7 days before induction of ulcer. Positive controls received omeprazole 30 mg/kg for the pylorus ligation-induced ulcer model and sucralfate 100 mg/kg for the acidified ethanol-induced ulcer model. Negative controls received vehicle (2% tween 80).

**Results:**

The crude hydromethanolic extract of *C. macrostachyus* showed significant (*p* < 0.05) antiulcer activity on both pyloric ligation and HCl/ethanol-induced ulcer in rats and mice. It has antisecretary effect (*p* < 0.001) as well. All three administered doses of chloroform fraction (*p* < 0.05) and only higher doses of ethyl acetate fraction (*p* < 0.05) possessed significant antiulcer activity. In contrast, the aqueous fraction did not have significant antiulcer effect at all tested doses.

**Conclusion:**

The present study demonstrated that the crude extract, chloroform, and ethyl acetate fractions possessed significant dose-dependent antiulcer activity.

## 1. Introduction

Globally, peptic ulcer disease (PUD) is the most common gastrointestinal disorder with significant mortality and morbidity [1]. It is a disease of the gastrointestinal tract (GIT), which includes both gastric and duodenal ulcers. It is characterized by imbalances between offensive (pepsin, gastric acid, and *Helicobacter pylori*) and defensive (prostaglandins, bicarbonate ions, mucin, growth factors, and nitric oxide) factors [2, 3].

With a high prevalence of *H. pylori* infection, morbidity and mortality of PUD continue to be increased [4]. It is also an important reason for hospitalization worldwide [5]. It claimed about 301,400 lives around the world in 2013 [6, 7]. Patients, who underwent surgery for PUD in Sub-Saharan Africa, indicated that 86% suffered from duodenal ulcers while the rest 14% had gastric ulcer. The major complications like perforation (35%), bleeding (7%), obstruction (30%), and chronic case (28%) were indicated for surgery, and the overall fatality rate was found to be 5.7% [8].

*H. pylori* and consumption of nonsteroidal anti-inflammatory drugs (NSAIDs) were common etiologic factors for PUD [9, 10]. Stress, tobacco smoking, alcohol intake, Zollinger Ellison syndrome, and age-related decline in prostaglandin levels are also mentioned as risk factors for PUD [11].

Currently used antiulcer drugs cause adverse reactions, such as hypersensitivity, arrhythmia, impotence, gynecomastia, hematopoietic changes, and kidney disease [10, 12]. These drugs also result in significant drug-drug interactions that limit the potential use of these agents [13, 14].

Traditional medicine plays a great role in providing primary health care worldwide [15]. About 75–80% people in developing countries use traditional medicine because of better cultural acceptability, better compatibility with the human body, and lesser side effects [10].

Large numbers of medicinal plants and their secondary metabolites have claimed antiulcer activity. Croton family was the source of many ingredients for the treatment of PUD in the world. An active ingredient, Plaunotol, was isolated from the stem bark and/or leaf of *Croton stellatopilosus.* Plaunotol induces PGE2, eradicates *H. pylori* bacteria, and used as a cytoprotective antiulcer agent. Plaunotol used in combination with clarithromycin and proton pump inhibitor was found to be more effective than other combinations for *H. pylori*-induced PUD [16, 17].

*C. macrostachyus* was the one with claimed antiulcer activity in Ethiopian traditional medicine, and around 83% of the respondents mentioned the antiulcer activity. The root, leaf, and bark decoction is also commonly used to treat abdominal pain, gastrointestinal disorders, and stomachache in tropical African countries [16, 18]. The local name for *C. macrostachyus* was bissana (Amharic) or bakanisa (Afan Oromo) [19, 20]. *C. macrostachyus* has other claimed medicinal uses in Ethiopia. The bark, fruits, leaves, roots, and seeds of this plant are traditionally used for treatment of diabetes, malaria, stomachache, cancer, ascariasis, abdominal pain, gonorrhea, wounds, ringworm infestation, hemorrhoids, and leishmaniasis [16, 21–24].

The root extract *C. macrostachyus* has good *in vitro* antibacterial (both Gram-negative and Gram-positive bacteria) [25] and antioxidant [20, 24] activities. A compound called crotepoxide was isolated from *C. macrostachyus* which was found to be active against *in vitro* leishmaniasis [25]. The *in vivo* antimalaria [26], antidiabetic [27], and anti-inflammatory [20] activities were also experimentally proved.

There are different types of *in vivo* experimental PUD models. Most of these models use rodent animals. Among these, ethanol-induced ulcer was used for the cytoprotective studies and the pyloric ligation-induced ulcer model for antisecretary and cytoprotective effects. Ethanol-induced ulcer resembles acute ulcer in humans and independent of gastric acid secretions. Depending the type of animal models used, positive control like sucralfate and Misoprostol are used for cytoprotective effect and drugs like H2 receptor blockers and proton pump inhibitors are used for the antisecretory effect [28].

Some secondary metabolites such as alkaloids, amino acids, anthraquinones, carbohydrates, cardiac glycosides, coumarins, essential oil, fatty acids, flavonoids, phenolic compounds, phlobatannins, polyphenols, phytosteroides, saponins, sterols, tannins, terpenoids, unsaturated sterol, vitamin C, and withanoides were reported in previous studies [24, 29].

Croton family is a major potential source for treatment of PUD. Among the Croton family, *C. macrostachyus* has proved *invitro* and *invivo* activities against different disease models due to the presence of important secondary metabolites. In Ethiopia, there is reported traditional claim of *C. macrostachyus* root for antiulcer effect. However, there was no scientific investigation made on the antiulcer activity on the root of the plant. Therefore, this study tries to evaluate the antiulcer activity of hydromethanolic crude extract and solvent fractions of the root of *C. macrostachyus* on rodent models.

## 2. Materials and Methods

### 2.1. Plant Source

The source of the sample was from the root of the wild plant, *C. macrostachyus.* The plant was authenticated by the botanist Dr. Getinet Masresha, and the voucher specimen (voucher no. = NA001) was deposited in the department of biology, University of Gondar, for future reference. Verbal permission was taken from the local community leader to use the plant. Since the sample was taken from some part of the source plant with care, it was left live, and hence, there was no probability for the plant to be damaged.

### 2.2. Collection of Plant Material

The root of *C. macrostachyus* was collected from Debre Libanos woreda, Central Ethiopia, on December 15, 2017 G.C. The root was dag out; washed with tap water to remove the soil and related impurities; and then sliced into small pieces and rapped with rubber plastic. This plant material was transported to the pharmacology laboratory, University of Gondar, and shade-dried at room temperature and crushed well with a miller.

### 2.3. Preparation of Hydromethanolic Root Extract

One kg of the coarsely powdered *C. macrostachyus* root was macerated with adequate amount of 80% methanol in an Erlenmeyer flask for 72 hours at room temperature. The extraction process was facilitated by occasional shaking. After 72 hours, the filtrate was first separated from the marc by using gauze and further filtered by using Whatman No. 1 filter paper. The marc was remacerated for another 72 hours twice, then filtered, and added to the first extract. After exhaustive extraction, the filtered extract was dried by using an oven set at 40°C followed by lyophilization to remove the remaining aqueous part.

From the crude extract obtained, 60 g was taken to undertake fractionation. It was separated into three discrete fractions in a standard procedure by distilled water, ethyl acetate, and chloroform using a separatory funnel. First, the crude extract was suspended in 360 ml distilled water. Then, equal volume of chloroform was added and vigorously shaken in a separatory funnel and left for 20 minutes until clear phase separation is formed. Since chloroform is denser than water, it is drained into a beaker by opening the control knob of the funnel. The chloroform fraction was dried by using an oven adjusted at a temperature of 40°C. In the same way, equal volume of ethyl acetate was added to the aqueous residue and vigorously shaken. Then, water was drained since it is denser than ethyl acetate. Ethyl acetate was collected and dried by using an oven adjusted at a temperature of 40°C. Water was removed by lyophilization from the aqueous fraction. This separation procedure by adding chloroform and ethyl acetate solvents was repeated three times.

### 2.4. Acute Toxicity Study

Acute toxicity study was conducted based on the limit test dose of 2000 mg/kg as described by OECD 425 guideline [30]. Three female Swiss albino mice were randomly grouped and kept in a cage. After being fasted for 2 hours, 2000 mg/kg of the extract dissolved in 2% tween 80 was administered to one mouse and observed for any signs of toxicity for 24 hours. On the next day, the remaining mice were administered 2000 mg/kg of the root extract and observed for any gross changes for 14 days.

### 2.5. Experimental Animals

Adult Sprague Dawley rats (150–200 g, 12–16 weeks) and Swiss albino mice (20–30 g, 10–14 weeks) of either sex, which were bred in the animal house of the Department of Pharmacology, University of Gondar, and purchased from the Ethiopian public health institution, were used for the experiment. The laboratory animals were healthy and drug-naive/test-free. The laboratory animals' welfare assessment was performed for gross physical changes, and those with wounds were excluded from the study. The animals were housed in polypropylene plastic cages with wood shavings as bedding material and maintained under standard conditions of light, humidity, and room temperature (19–25°C; 12 hrs light and dark cycles). Standard pellet and tap water were provided *ad libitum*.

### 2.6. Grouping of Animals and Dosing

For the pylorus ligation-induced ulcer model, thirty Sprague Dawley rats of either sex (150–200 g) were randomly divided into five different groups of six rats in each group. The negative control (NC) group was given 2% tween 80 (Group I). Treatment groups II, III, and IV were given *C. macrostachyus* crude extract of 100 mg/kg (CM100), 200 mg/kg (CM200) and 400 mg/kg (CM400), respectively. Group V was treated with a 30 mg/kg omeprazole (OM30). The given doses were determined based on the safety of the *C. macrostachyus* in acute toxicity study.

For HCl/ethanol-induced ulcer model, thirty Swiss albino mice (20–30 g) of either sex were randomly divided into five groups of 6 mice in each group. Negative control (NC) and treatment groups for crude extract were similar to those of the pylorus ligation-induced ulcer model. However, the standard drug used for the HCl/ethanol-induced ulcer model was 100 mg/kg sucralfate (SUC100).

For fractions, the HCl/ethanol-induced ulcer model was used. Sixty-six Swiss albino mice (20–30 g) of either sex were randomly divided into eleven groups of 6 mice in each group (*n* = 6). Negative control (NC) was given with 2% tween 80 (Group 1). Groups II, III, and IV were treated with 100 mg/kg (CF100), 200 mg/kg (CF200), and 400 mg/kg (CF400) chloroform fractions, respectively. Groups V, VI, and VII were treated with 100 mg/kg (EF100), 200 mg/kg (EF200), and 400 mg/kg (EF400) ethyl acetate fractions, respectively. Groups VIII, IX, and X were treated with 100 mg/kg (AF100), 200 mg/kg (AF200), and 400 mg/kg (AF400) aqueous fractions, respectively. Group XI was treated with 100 mg/kg sucralfate (SUC100). All treatments were given PO, for 7 days, and between 9 and 10 am in the morning for pyloric ligation- and HCl/ethanol-induced ulcer models.

### 2.7. The Pyloric Ligation-Induced Ulcer Model

The method proposed by Shay et al. was used with slight modification. The rats were deprived of food for 1 day before pyloric ligation, but had free access to water. The rats were put with wide-mesh wire bottoms to prevent coprophagia during the experiment. Pylorus ligation was performed in all rats for the induction of gastric ulcers 1 h after the last administration of the respective test solutions on fasted rats. The abdomen was opened by using a small incision below the xiphoid process after induction of anesthesia by ketamine HCl (75 mg/kg, intraperitonial). The stomach was exposed, and a thread was placed around the pyloric sphincter and tied in a tight knot. Care had been taken while tying the knot to avoid damage of involving blood vessels in the knot. The abdomen was sutured, and the animals were sacrificed 4 hrs after euthanasia by overdosing with an anesthetic agent (halothane). The stomachs were removed, and the contents were drained into tubes and centrifuged at 1000 rpm for 10 minutes. The supernatant was then subjected to analysis for gastric volume, pH, and acidity [31].

#### 2.7.1. Macroscopic Evaluation of Stomach

The stomachs were then cut along the greater curvature, rinsed with normal saline to remove gastric contents, and examined by using a 10x magnifier lens to assess the formation of ulcers. The numbers of ulcers were counted and then scored by using the Kulkarni method (0 = no ulcer, 0.5 = red coloration, 1 = spot ulcers, 2 = deep ulcers, and 3 = perforations) [32]. This procedure was performed by an expert in identification of ulcer types. The expert was blinded for the source of animal groups. The ulcer index and percentage of ulcer inhibition were determined as follows:

(1) $$\begin{array}{c} \text{ulcer index }\left(\text{UI}\right)\;=\;U_{\text{N}}\;+\;U_{\text{S}}+\;U_{\text{P}}{\;\times \;10}^{-1}, \end{array}$$

where *U*_N_ = average number of ulcers per animal, *U*_S_ = average of severity score, and *U*_P_ = percentage of animals with ulcers.

(2) $$\begin{array}{c} \text{ulcer inhibition }\left(\text{\%}\right)\;=\left(\frac{\left({\text{ UI}}_{\text{control}}-{\text{UI}}_{\text{pretreated}}\right)}{{\text{ UI}}_{\text{control}}}\right)\;\times \;100\text{ acidity}\left(\text{mEg}/\text{L}\right)=\frac{\left(\text{VNaOH }\times \text{N }\times 100\text{mEq}/\text{L}\right)}{0.1}\text{.} \end{array}$$

#### 2.7.2. Determination of Gastric Volume and pH

The contents were drained into tubes and centrifuged at 1000 rpm for 10 minutes, and the volume was noted. Then, an aliquot of 1 ml gastric juice was diluted with 1 ml of distilled water, and pH of the solution was measured using a pH meter.

#### 2.7.3. Determination of Total Acidity

An aliquot of 1 ml gastric juice diluted with 1 ml of distilled water was taken into a 50 ml conical flask, and two drops of phenolphthalein indicator was added to it and titrated with 0.01 N NaOH until a permanent pink color was observed. The volume of 0.01 N NaOH consumed was noted. The acidity was expressed as mEq/L by the following formula:

(3) $$\begin{array}{c} \mathrm{acidity}\;\left(\text{mEq/L}\right)\;=\;V_{\mathrm{NaOH}\;}\times \;N\;\times \;100\text{ mEq/L,} \end{array}$$

where *V* = Volume and *N* = Normality.

#### 2.7.4. Determination of Gastric Mucus Content

The procedure by Corne et al. was used to determine gastric-wall mucus with slight modification. The glandular segments from the stomach were removed and weighed. Each segment was transferred immediately to 10 ml 0.1% alcian 8GX blue solution (in 0.16 M sucrose solution, buffered with 0.05 M sodium acetate, and adjusted to pH 5.8) for 2 hrs, and the excess dye was removed by two successive rinsing for 15 and 45 minutes with 0.25 M sucrose solution. The dye complexed with the gastric-wall mucus was then extracted with 20 ml of 0.05 M magnesium chloride for 2 hrs with occasional shaking. A 5 ml sample of blue extract was then shaken with an equal volume of diethyl ether for 2 hrs with an interval of 30 min. The resulting emulsion was centrifuged at 3000 rpm for 15 minutes, and the absorbance of the aqueous layer was measured and recorded at 580 nm using a UV-Visible spectrophotometer. The quantity of alcian blue extracted/g (net) of glandular tissue was then calculated from the standard curve of alcian blue. Taking 20, 25, 30, 40, 50, 60, 70, and 80 *μ*g/10 ml of alcian blue solutions, the absorbance of each solution was measured and the graph was made for absorbance (*Y*) versus concentration (*X*) to be taken as the standard graph of alcian blue solution [33].

### 2.8. Acidified Ethanol-Induced Ulcer Model

The procedure was carried out according to Mizui et al. with slight modifications. The mice were deprived of food overnight before the experiment, but had free access to water. The mice received the crude extract and solvent fractions treatment 60 minutes before the administration of acidified ethanol solution (150 mM HCl/ethanol, 40 : 60 v/v). One hour after the administration of acidified ethanol, the mice were euthanatized by overdosing with an anesthetic agent (halothane) and sacrificed by cervical dislocation. The stomachs were excised and gently rinsed with normal saline, inflated with 1% formalin solution (10 ml), and immersed in the same solution to fix the outer layer of the stomach [34]. After 10 min, each stomach was then opened along the greater curvature and examined under a dissecting microscope to assess the formation of ulcers, then scored, and the ulcer index (UI) was calculated and percentage of ulcer inhibition was determined as described in the pyloric ligation model [31].

### 2.9. Ethical Statement

All the experiments were conducted in accordance with the guide for the care and use of laboratory [35]. At the end of each experiment, the rats were sacrificed with high dose of halothane, and the mice were sacrificed by cervical dislocation. Ethical clearance was obtained from the research and ethics committee, department of pharmacology, University of Gondar, with Reference number of SOP 4/50/10 to conduct the study in animal models. Apart from that, all possible steps were taken to avoid animal suffering at each stage of the experiment.

### 2.10. Data Analysis

Data were entered and analyzed using SPSS version 20 statistical software. The values are expressed as Mean ± SEM. One-way ANOVA followed by the post hoc Tukey test was employed for statistical comparison across groups. At 95% confidence interval, *p* < 0.05 was considered statistically significant.

## 3. Results

### 3.1. Crude Extract and Fractionation

From crude extract, the percentage yield was 9.4%. For fractions, the yields of chloroform, ethyl acetate, and aqueous fractions were found to be 13%, 3.1%, and 83.8% respectively.

### 3.2. Acute Oral Toxicity Study

The acute toxicity study indicated that the root extract caused no mortality at 2000 mg/kg within the first 24 hours and for the next 14 days. Physical and behavioral observations of the experimental mice also revealed no visible signs of acute toxicity such as lacrimation, loss of appetite, tremors, hair erection, salivation, and diarrhea.

### 3.3. Effects of Crude Extract on Pyloric Ligation-Induced Ulcer in Rats

**Figure 1:**
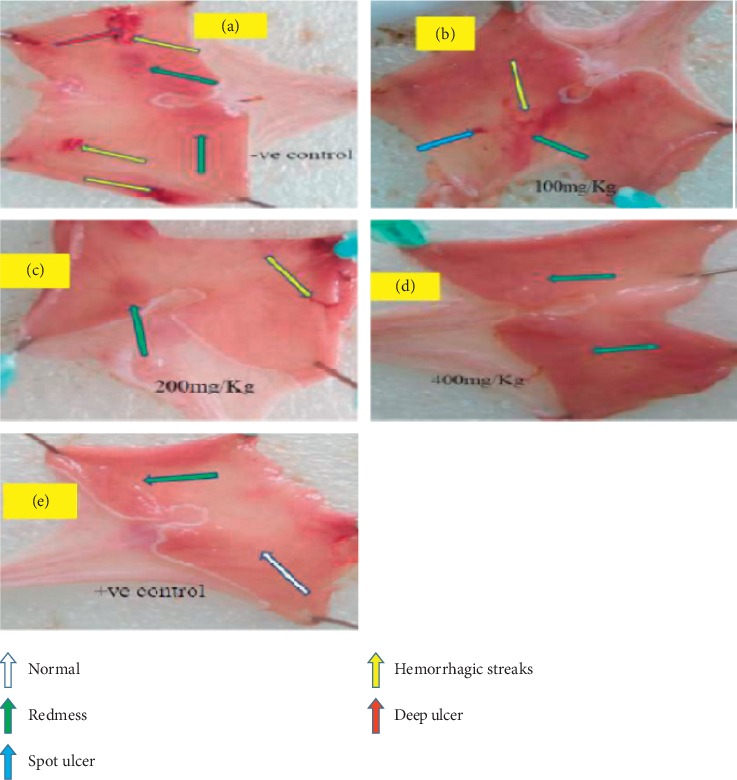
Effect of crude extract of *C. macrostachyus* on the pylorus ligation-induced ulcer model in rats. a = NC, b = CM100, c = CM200, d = CM400, e = OM30. In this figure, we can see that deep ulcer and hemorrhagic streaks were observed in the negative control (a) and some hemorrhagic streaks and minor spots were also observed in the lower dose of the extract (b). As we go in advance from lower dose to higher (b-c, d), it showed that significant ulcer formations were not observed.

The pyloric ligation procedure caused the accumulation of gastric secretions. All the animals were included in for the analysis (6/6) for all procedures. There was minor bleeding during the pylorus ligation procedure which was stopped by holding with cotton immersed in normal saline. This bleeding was absent as we perform more pyloric ligation procedures. The CM200 and CM400 pretreated groups have significantly lowered gastric volume as compared to that of CM100 and NC (*p* < 0.001 for both cases). CM400 (21.50 ± 0.43 ml) has comparable activity regarding gastric volume reduction with OM30 (19.83 ± 0.60 ml). OM30 pretreated rats has lower gastric volume as compared to that of CM200 (*p* < 0.05) and CM100 (*p* < 0.001). In our study, gastric acidity in CM200 and CM400 pretreated groups was lowered than that of NC and CM100 pretreated groups (*p* < 0.001 for both cases). CM100 pretreated groups showed no acid reducing activity as compared to that of NC. In terms of pH, CM200 and CM400 significantly elevated the pH as compared to that of NC and CM100 (*p* < 0.001 for both cases). Furthermore, it was observed that pyloric ligation has caused gastric ulceration and pretreatment with the root extract of *C. macrostachyus* has lowered gastric ulceration in a dose-dependent manner (Figure 1). The CM400 pretreated group had significantly lowered gastric ulceration as compared to NC (*p* < 0.001) and CM100 (*p* < 0.05). The CM200 pretreated group had less gastric ulceration as compared to that NC (*p* < 0.05) (Table 1).

The gastric mucus content was found to be 73.97 ± 0.36 *μ*g/g of tissue from the NC group. Pretreatment with CM200 and CM400, however have significantly higher (*p* < 0.001) gastric mucus levels. The average mucus content for CM200 and CM400 groups was 107.31 ± 0.58 and 173.00 ± 0.28 *μ*g/g of tissue, respectively. The gastroprotection activity offered by the test extract of CM200 and CM400 was comparable to that of OM30 pretreated group while CM100 pretreated group showed comparable mucus levels to that of NC (Table 1).

In pyloric ligation model, percentage inhibition of ulceration was found to be 63.9% and 37.7% in CM400 and CM200 pretreated groups, respectively. The standard drug OM30 also decreased the ulcer formation by 72.9% which is comparable with that of CM400 (data not shown).

### 3.4. Effects of Crude Extract on Acidified Ethanol-Induced Ulcer in Mice

**Figure 2:**
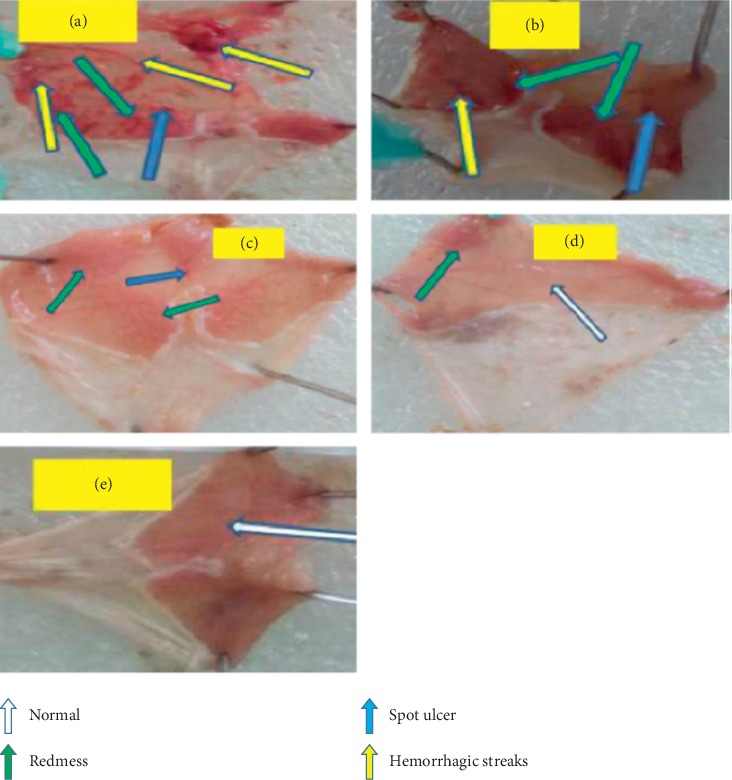
Effect of crude extract of *C. macrostachyus* on the acidified ethanol-induced ulcer model in mice. a = NC, b = CM100, c = CM200, d = CM400, and e = OM30. In this figure, a spot ulcer and many hemorrhagic streaks were observed in the negative control (a), and some hemorrhagic streaks and minor spots were also observed in the lower dose of the extract (B). As we go in advance from lower dose to higher doses (b-c, d), it showed that significant ulcer formations were not observed as compared to the negative control.

The acidified ethanol (0.15 M HCl/ethanol) at dose of 5 ml/kg showed superficial ulcer and hemorrhagic streak formation in the control animals. However, animals pretreated with CM200 (*p* < 0.05) and CM400 (*p* < 0.001) doses showed significantly lowered number of ulcers and ulcer index as compared to those of NC (Figure 2). It showed 45.9% and 62.2% ulcer inhibition at the dose of CM200 and CM400, respectively, whereas sucralfate showed 80% ulcer inhibition. Antiulcerogenic effect of the CM400 pretreated animals was comparable to that of standard drug, SUC100, while CM100 showed minimal antiulcerogenic effect (Table 2).

### 3.5. Effects of Fractions on Acidified Ethanol-Induced Ulcer in Mice

**Figure 3:**
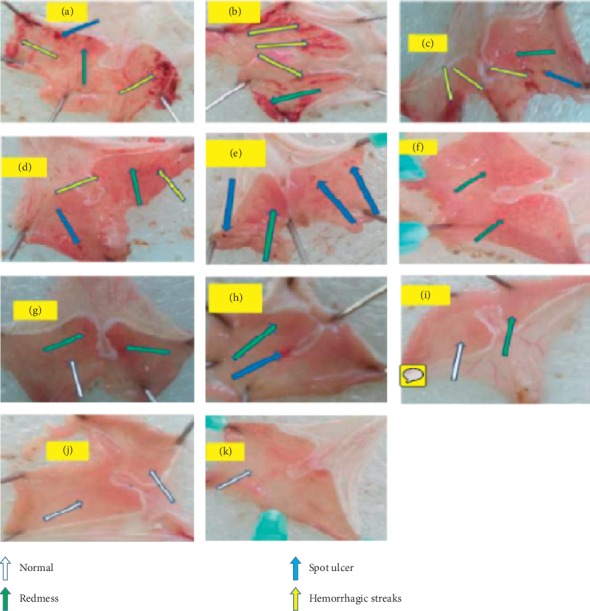
Effects of fractions of *C. macrostachyus* on the acidified ethanol-induced ulcer model in mice. a = NC, b = AF100, c = AF200, d = AF400, e = EF100, f = EF200, g = EF400, h = CF100, i = CF200, j = CF400, and k = SUC100. In this figure, some spot ulcers and many hemorrhagic strikes were obviously observed from the negative control (a) and almost in all doses of aqueous fractionate (b, c, d). On the other hand, as we go from ethyl acetate fractionate to chloroform fractionate (e, f, g and h, i, j; from lower to higher doses, respectively), the formation of different types of significant ulcers were not well-observed in advance.

In the acidified ethanol-induced ulcer model, chloroform fraction pretreated groups significantly prevented ulcer formation (CF100 (*p* < 0.05), CF200 (*p* < 0.001), and CF400 (*p* < 0.001)) compared to that of NC. The standard drug, SUC100, does not show any significant variation in ulcer index as compared to all doses of chloroform fractions. Similarly, higher doses ethyl acetate fractions EF200 (*p* < 0.05) and EF400 (*p* < 0.001) significantly prevented the occurrence of ulcer formation as compared to that of NC. The standard drug, SUC100, showed comparable activity with EF200 and EF400. On the contrary, the EF100 pretreated animals does not show any significant variation in ulcer index as compared to that of NC (Figure 3). All tested doses of the aqueous fraction showed no significant antiulcer activity as compared to NC (Table 3).

**Figure 4:**
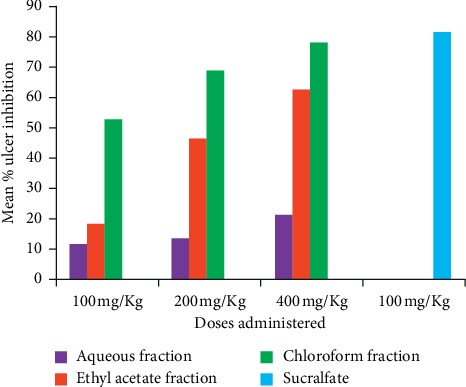
Percent ulcer inhibition of fractionations of *C. macrostachyus* on the acidified ethanol-induced ulcer model in mice.

Comparison of the similar doses of chloroform and ethyl acetate fractions showed higher percentage inhibition activity from chloroform fraction (CF100 > EF100; CF200 > EF200; and CF400 > EF400) (Figure 4). Percent protection of fractions of *C. macrostachyus* root indicated that the ulcer protection capacity of chloroform and ethyl acetate fractions increased significantly as dose increases from lower to higher while the aqueous fraction did not.

## 4. Discussion

In the present study, the percentage yield was 9.4% which is comparable to 10% yield of the previous study [26]. The results showed that there was a significant decrease in gastric acidity and stomach secretion, a significant increase in pH and mucus content by pretreatment of the crude extract of *C. macrostachyus* root on pyloric ligation-induced ulcer in rats. Moreover, there was a significant decrease in ulcer formation in both pyloric ligation and HCl/ethanol-induced ulcer models in a dose-dependent manner.

An ulcer can be induced by different mechanisms. The most commonly used method for induction of ulcer was pyloric ligation. Pyloric ligation will result in accumulation of gastric acid and activation of pepsin. These in turn will result in formation of ulcer [28]. Mucosal digestion also decreases the synthesis of prostaglandin E2 and I2 which are important factors for the inhibition of gastric acid secretion, stimulation of mucus, bicarbonate, and phospholipids secretion in the gastric epithelial cells [36]. There is also the involvement of histamine in the formation of pyloric ligated ulcers [37].

In previous studies, the root extract of *C. macrostachyus* contained secondary metabolites like phenolic compounds, tannins, flavonoids, coumarins, saponins, and polyterpenes [26]. Some phytoconstituents extracted from medicinal plants possess antiulcerogenic activity and act by various mechanisms. Phenolic compounds and flavonoids possess antiulcer effect due to their antisecretory, cytoprotective, antioxidant, anti-inflammatory, and anti-*H. pylori*. Phenolic compounds and flavonoids also promote prostaglandin synthesis, stress defense, and antioxidant enzymes synthesis, and wound healing properties [38–42]. Moreover, flavonoids increase capillary resistance and improve microcirculation. Tannins directly protect the outermost layer of mucosa and change the mucosa structure that can resist to chemicals and mechanical injury [43]. Saponins- and triterpenoid-related compounds increase mucus production [44]. As a result, the significant antiulcer effect of the crude extract of the root of *C. macrostachyus* on mucus production may be associated with the presence of active secondary metabolites like phenolic compounds, tannins, flavonoids, saponins, and polyterpenes.

Various mediators are directly and indirectly involved in noxious function of ethanol such as leukotriens, cytokines, and oxygen-derived free radicals. The HCl/ethanol-induced gastric ulcer model was used because it is relatively similar to that observed in acid hypersecretion cases in humans. Ethanol indirectly damages cell membrane of the gastric epithelium via increased lipid peroxidation and directly damages superficial mucosal cells [45]. The addition of HCl causes acceleration of the ulcerogenesis process, intensifies injuries, and reduces the mucosal protection against chemical agents [46]. We can make sure the formation of ulcer by acidifying the ethanol. Ethanol by itself does not affect the secretion of gastric acid [28]. Ethanol also results in an increased production of oxygen free radicals within the tissues when metabolized in the body, which simultaneously increases the cellular free radical concentration. These free radicals caused damages of DNA strands and protein denaturation [47]. Free radicals also deplete prostaglandin levels there by inhibiting gastric mucus and bicarbonate secretion and stimulating acid secretion [48]. The efficacy of hydromethanolic extract of *C. macrostachyus* (*p* < 0.05) in the HCl/ethanol-induced gastric ulcer model might be the cytoprotective or the antioxidant effect *C. macrostachyus*. The antioxidant and free radical scavenging properties of the crude extract of *C. macrostachyus* root were reported in the previous studies [29, 49]. This result was in agreement with the previously conducted similar studies in Croton species [50].

The effects of fractionations were also investigated in this study on the HCL/ethanol-induced ulcer model in mice. All tested doses of chloroform fraction had significant antiulcer activity (*p* < 0.05) which is in agreement with the previous study in *Bauhinia purpurea* leaf [36]. Similarly, higher doses of ethyl acetate fraction also showed significant antiulcer activity (*p* < 0.05) which is in line with the previous study conducted on *Merremia tridentata* [51]. On the other hand, all tested doses of aqueous fraction were devoid of significant antiulcer activity. Lack of activity from aqueous fraction and need for higher doses from ethyl acetate fraction shows that there might be large accumulation of active secondary metabolites in the nonpolar solvents. In this case, the concentration of active secondary metabolites with antiulcer activity might be higher in chloroform fraction followed by ethyl acetate fraction while smaller in case of aqueous fraction.

A study conducted on the root extract analysis of *C. macrostachyus* showed that the chloroform fraction (IC_50_ = 35.45 *μ*g/ml) and ethyl acetate fraction (IC_50_ = 88.79 *μ*g/ml) have free radical scavenging activity [29]. Based on their IC_50_, the chloroform fraction was effective at lower concentration as compared to ethyl acetate. This may, at least, be one of the reasons why chloroform fraction of *C. macrostachyus* showed antiulcer activity at all given doses while ethyl acetate fraction showed activity only at medium and high doses suggesting the relatively higher potency of chloroform fraction. Therefore, the plausible mechanism of action of the fractions could be free radical scavenging and increasing blood flow of the stomach thereby increasing the protective lining and mucus production.

Pain and stress should be reduced before, during, and after the pylorus ligation procedure because in mammals and humans, stress caused by different type of pain can activate corticotrophin releasing factor-1 receptors, leading to increase in acid secretion and the development of gastric ulcer [52]. Hence, use of anesthetics like ketamine can decrease the pain and stress caused by the pyloric ligation procedure [53].

In the present study, we used the Kulkarni method of ulcer index determination that includes number and severity of ulcer [32]. However, this method has some limitations, as it did not incorporate the total area of stomach in determining the ulcer index. This may produce a statistically correct ulcer index but may fail to have a biologically relevant ulcer.

## 5. Conclusions

In summary, the present study showed that the hydromethanolic root extract of *C. macrostachyus* has significant antiulcer activity which upholds the traditional claim of the experimental plant. From fractionation tests, the chloroform fraction was found to be more effective while the aqueous fraction lacks antiulcer activity. The isolation and structural elucidation of active compounds in the chloroform fraction and *in vitro* activity against *H. pylori* should be tested. The antiulcer activity along with its safety profile could make the root of *C. macrostachyus* a good candidate for the treatment of PUD in humans.

## Figures and Tables

**Table 1:** Antiulcer activity of crude extract of the root of *C. macrostachyus* on the pyloric ligation ulcer model in rats.

Group	Ulcer index	pH	Acidity in mEq/L	Gastric volume (ml)	Mucus content (*μ*g/g)
NC	21.25 ± 1.7^c1d3e3^	2.08 ± 0.17^c3d3e3^	60.50 ± 0.76^c3d3e3^	3.77 ± 0.14^c3d3e3^	73.97 ± 0.36^c3d3e3^
CM100	16.92 ± 1.29^d1e2^	2.55 ± 0.09^c3d3e3^	58.33 ± 0.42^c3d3e3^	3.40 ± 0.06^c3d3e3^	75.75 ± 0.41^c3d3e3^
CM200	13.25 ± 0.72^a1^	3.72 ± 0.12^a3b3d3e3^	46.00 ± 0.58^a3b3d3e3^	2.63 ± 0.07^a3b3d2e3^	107.31 ± 0.58^a3b3d3e3^
CM400	7.67 ± 2.42^a3b1^	5.53 ± 0.07^a3b3c3^	21.50 ± 0.43^a3b3c3^	2.07 ± 0.08^a3b3c2^	173.00 ± 0.28^a3b3c3^
OM30	5.75 ± 2.57^a3b2^	5.83 ± 0.13^a3b3c3^	19.83 ± 0.60^a3b3c3^	1.83 ± 0.12^a3b3c3^	174.62 ± 0.67^a3b3c3^

Values are expressed as Mean ± SEM, *n* = 6, ^1^*p* < 0.05, ^2^*p* < 0.01, ^3^*p* < 0.001, ^a^compared to NC, ^b^compared to CM100, ^c^compared to CM200, ^d^compared to CM400, and ^e^compared to OM30. Statistically analyzed by one-way ANOVA followed by the Tukey test.

**Table 2:** Antiulcer activity of crude extract of the root of *C. macrostachyus* on acidified ethanol-induced ulcer model in mice.

Groups	Average ulcer number (UN)	Ulcer index	% of ulcer inhibition
NC	5.33	22.50 ± 1.95	—
CM100	3.33	17.00 ± 1.07^e2^	24.44
CM200	2.17	12.17 ± 0.38^a1^	45.93
CM400	0.83	8.50 ± 2.80^a3^	62.22
SUC100	0.67	7.04 ± 2.87^a3b2^	80

Values are expressed as Mean ± SEM, *n* = 6, ^1^*p* < 0.05, ^2^*p* < 0.01, ^3^*p* < 0.001,^a^compared to NC, ^b^compared to CM100, ^c^compared to CM200, ^d^compared to CM400, and ^e^compared to SUC100. Statistically analyzed by one-way ANOVA followed by the Tukey test.

**Table 3:** Effects of the fractions of the *C. macrostachyus* root extract on the acidified ethanol induced ulcer model in mice.

Groups	Dose	Average ulcer no. (UN)	Ulcer index (UI)	% of ulcer inhibition
I	NC	4.33	20.58 ± 1.27	--—
II	CF100	1.5	9.67 ± 3.07^a1^	53.0
III	CF200	0.83	6.42 ± 2.89^a2^	68.8
IV	CF400	0.67	4.50 ± 2.89^a3^	78.1
V	EF100	3.33	16.75 ± 0.80^d1e3^	18.6
VI	EF200	1.5	11.00 ± 2.29^a1^	46.6
VII	EF400	0.67	7.66 ± 2.42^a2b1^	62.8
VIII	AF100	3.83	18.17 ± .65^e3^	11.7
IX	AF200	3.67	18.25 ± 0.48^e3^	11.32
X	AF400	3.17	16.17 ± 1.48^e3^	21.4
XI	SUC100	0.33	3.83 ± 2.42^a3b3c3d3^	81.4

Values are expressed as Mean ± SEM, *n* = 6, ^1^*p* < 0.05, ^2^*p* < 0.01, ^3^*p* < 0.001, ^a^compared to NC, ^b^compared to 100 mg/kg, ^c^compared to 200 mg/kg, ^d^compared to 400 mg/kg, and ^e^ compared to SUC100. Statistically analyzed by one-way ANOVA followed by the Tukey test.

## Data Availability

The datasets analyzed during the current study are available from the corresponding author on reasonable request.

## Abbreviations

GIT: Gastrointestinal tract
NC: Negative control
OECD: Organization for Economic Cooperation and Development
PO: Per oral
PUD: Peptic ulcer disease.
